# Primary Psychiatric Disorder Masking the Diagnosis of Neuropsychiatric Lupus in a Patient with Altered Mental Status: A Case Report

**DOI:** 10.7759/cureus.1793

**Published:** 2017-10-23

**Authors:** Osman Perez, Kairavee Dave, Aimee Almanzar, Tajul Prodhan, Livasky Concepion

**Affiliations:** 1 GME, Aventura Hospital and Medical Center; 2 Internal Medicine, Aventura Hospital and Medical Center

**Keywords:** neuropsychiatric systemic lupus erythematosus, npsle, lupus, altered mental status, neuropsychiatry, gamma gap, lupus cerebritis

## Abstract

Neuropsychiatric systemic lupus erythematosus (NPSLE) has a wide variety of neurologic and psychiatric features. NPSLE symptoms and the psychotic features of primary psychiatric disorders often overlap with each other. These psychotic features often mask and delay the diagnosis of NPSLE. We present the case of a 59-year-old female previously diagnosed with bipolar disorder and generalized anxiety disorder presenting with altered mental status (AMS), subsequently diagnosed with neuropsychiatric lupus.

Initially, medication overdose was suspected as an empty bottle of trazodone was found beside her. Obtaining an appropriate history was difficult due to the patient’s altered mental status and absence of family members at bedside. The patient was found to have an elevated gamma gap, and further workup was pursued. Subsequently, positive antinuclear antibody (ANA) and anti-double stranded DNA antibody (anti-dsDNA) was detected. During the hospitalization, she was found to meet the Systemic Lupus Erythematosus International Collaborating Clinics (SLICC) criteria for systemic lupus erythematosus (SLE). Lumbar puncture with cerebrospinal fluid (CSF) analysis revealed lymphocytic pleocytosis, elevated protein with no bacteria and likely a non-infectious process. Magnetic resonance imaging (MRI) spectroscopy of the brain revealed a reversal of normal Hunter's angle, with elevated choline-to-creatine ratio within the white matter, and a lactate peak, which may be present in neuropsychiatric lupus. The patient was diagnosed with SLE with neuropsychiatric manifestations. Consequently, a kidney biopsy was obtained showing Class IV diffuse proliferative glomerulonephritis with fibrillary component likely related to lupus nephritis. The patient was started on treatment for neuropsychiatric lupus, which includes treatment for lupus nephritis with high dose pulse methylprednisolone. The anti-dsDNA titers decreased from 81 to 15 IU/ml and the patient displayed a gradual improvement in her mental status. She was started on cyclophosphamide while inpatient and discharged with the combination of cyclophosphamide, prednisone, along with rheumatology follow-up.

This case stresses the importance of ruling out organic causes of AMS before diagnosing patients with a psychiatric disorder. Not every patient with SLE will meet the criteria for diagnosis at the same point in time; hence, it is important to obtain an appropriate history and physical examination to support such diagnosis. We believe our patient had a neuropsychiatric manifestation of SLE, which demonstrates the importance to keep this diagnosis in the list of differentials when assessing a patient presenting with AMS.

## Introduction

Systemic lupus erythematosus (SLE) is a chronic autoimmune inflammatory condition with a spectrum of clinical presentations. Neuropsychiatric systemic lupus erythematosus (NPSLE) is often a diagnostic challenge because of its overlapping features with primary psychiatric disorders. The psychotic features may primarily originate from SLE, complications of this disease, or as a result of the recommended therapy. Here, we present a case of a 59-year-old female who was initially diagnosed with primary psychiatric disorder, later found to have neuropsychiatric lupus.

## Case presentation

A 59-year-old female with past medical history of bipolar disorder and anxiety, previously diagnosed at our facility and under treatment, presented with sudden onset of altered mental status. According to police records, she was found near an empty bottle of trazodone and acetaminophen/butalbital/caffeine. When her family became available, they described the patient as confused, disoriented, mumbling words out of context to the conversation, which made the initial case for drug overdose. A few months prior, and under similar circumstances, she was found to be unresponsive at home requiring admission to the critical care unit for cardiorespiratory support. In addition, she had presented to emergency rooms (ER) multiple times with anxiety and panic attacks requiring psychiatric evaluation and behavioral management including benzodiazepines, anxiolytics, and antidepressants. In this occasion, the patient was found to be disoriented, answering in complete sentences, but incoherently. Muscle bulk and tone was normal with no apparent weakness. Neurological examination was limited due to altered mental status; however, no neurological deficits, neck stiffness or papilledema were noted on physical examination. She was treated acutely with activated charcoal for possible drug overdose.

Initial differential diagnoses were broad, including medication overdose, stroke, sepsis, hypovolemia, hypoglycemia, and electrolyte imbalances. An extensive workup was done and most of the initial differential diagnoses were excluded. However, the patient was found to have an elevated gamma gap, elevated blood urea nitrogen (BUN) and creatinine, nephritic-range proteinuria, and anemia, which prompted a rheumatologic workup. Antinuclear antibody (ANA) and anti-double stranded DNA antibody (anti-dsDNA) were found to be positive which lead to a nephrology evaluation. NPSLE was then suspected.

**Figure 1:**
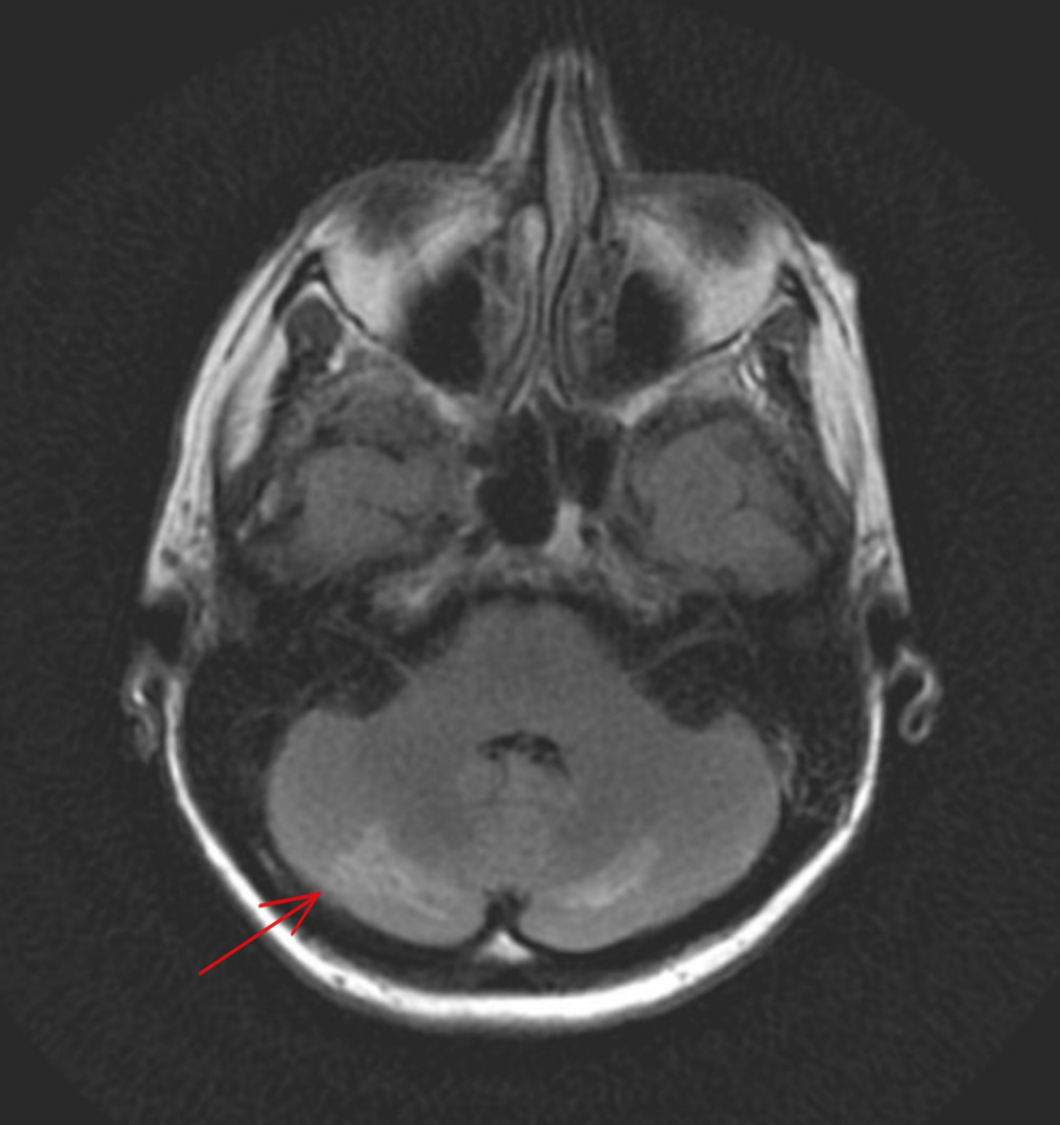
Magnetic resonance imaging (MRI) of the brain without contrast showing focal cerebellar enhancement.

A head computed tomography (CT) revealed hypodensities along the bilateral posterior cerebellar peduncles and bilateral posterior cerebellar hemispheres. Lumbar puncture produced a clear cerebrospinal fluid, with lymphocytic pleocytosis, elevated protein, and normal glucose, consistent with aseptic meningitis. Magnetic resonance imaging (MRI) of the brain with contrast showed patchy focal enhancements and areas of signal abnormality in the posterior cerebellar hemispheres (Figure 1). Electroencephalogram showed moderate cerebral dysfunction without definitive seizure pattern. MRI spectroscopy revealed a reversal of normal Hunters’ angle with elevated choline-to-creatine ratio within the white matter of right and left cerebral hemispheres, which reflects the cerebral metabolic disturbance found in patients with neuropsychiatric lupus.

**Figure 2:**
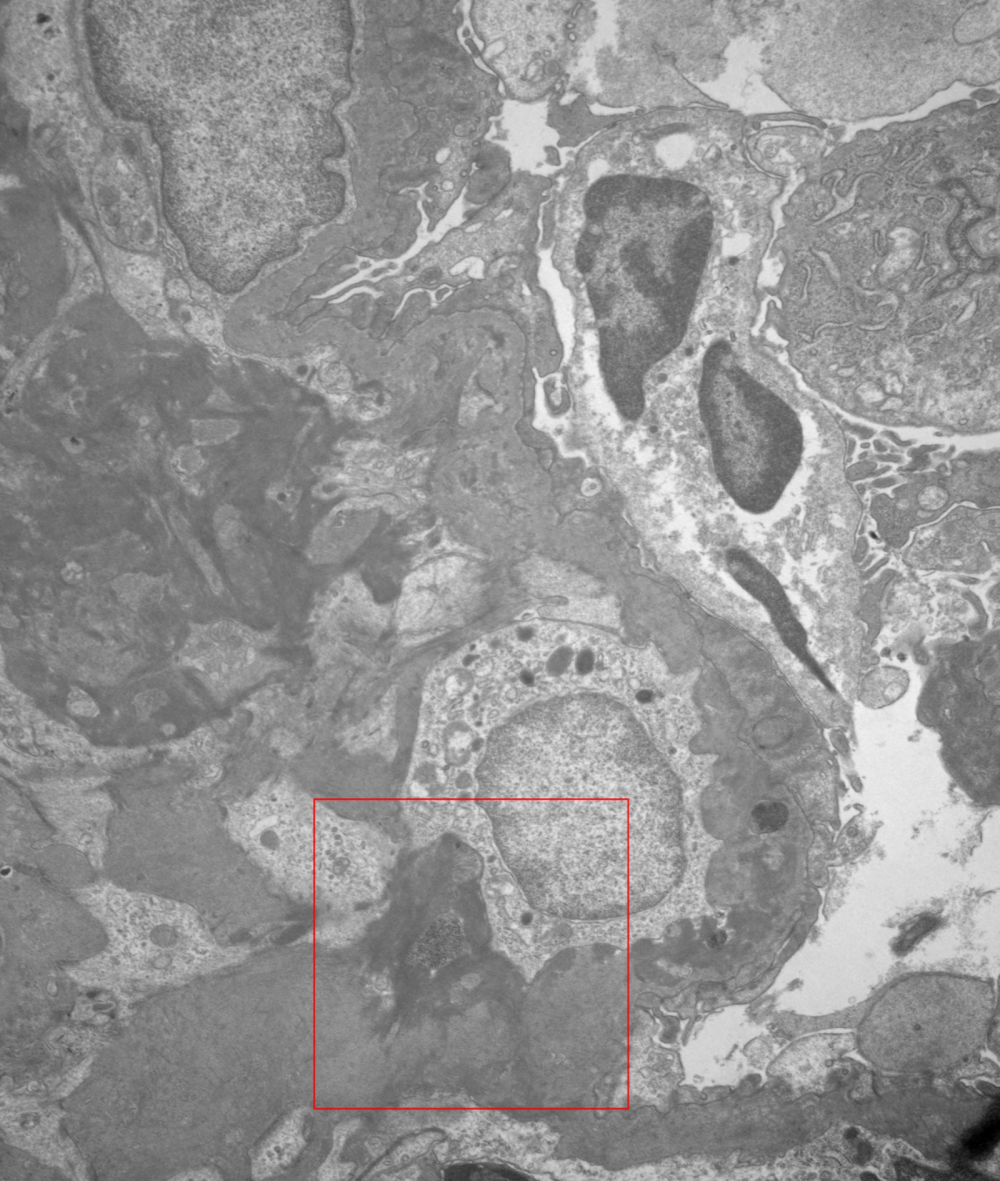
Electron microscopy of the kidney showing thickened basement membrane.

**Figure 3:**
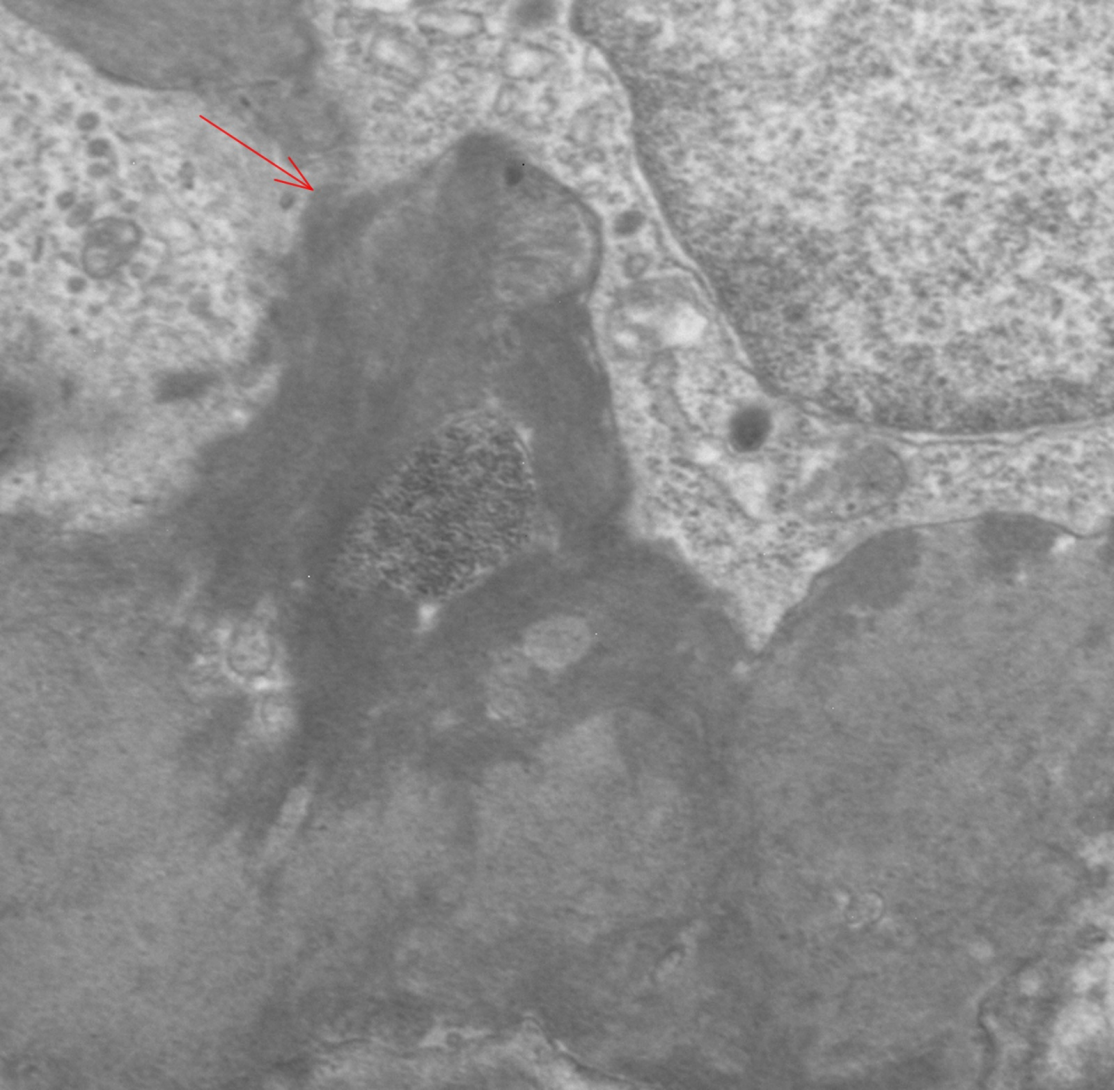
Electron microscopy of the kidney detailing fibrillary components.

A diagnosis of NPSLE was made in view of acute confusional state with abnormal MRI findings, aseptic meningitis, positive ANA, anti-dsDNA in fulfillment of Systemic Lupus Erythematosus International Collaborating Clinics (SLICC) criteria. Subsequently, kidney biopsy was done due to significant proteinuria. Biopsy showed class IV diffuse proliferative glomerulonephritis with thickened basement membrane and fibrillary components suggestive of lupus nephritis (Figures 2, 3). Intravenous high-dose methylprednisolone was initiated which reduced the anti-dsDNA titer from 81 to 15 IU/ml over 10 days. Her mental status also began to show signs of improvement. Prior to discharge, she was commenced on cyclophosphamide along with a weaning regimen of oral prednisone, IV cyclophosphamide monthly for a total of six doses. She was discharged to a skilled nursing facility for intravenous drug administration, and a decline in her activities of daily living. Rheumatology continued to follow the patient in the outpatient setting.

## Discussion

The prevalence of SLE is approximately 130/100,000 in the United States, affecting African Americans, Hispanics, and Asians more frequently than Non-Hispanic Whites. Additionally, it is nine times more prevalent in women than in men [6].

On the other hand, the prevalence of NPSLE has been more difficult to quantify since it varies according to the criteria used for diagnosis. As an example, Faria, et al. reported a prevalence of 24%, based on the SLICC initiative NPSLE cohort. Interestingly, the initial value was 87.6%, however, it dropped significantly after the exclusion of patients with characteristics that would make the diagnosis of NPSLE less likely. These included (a) neuropsychiatric event taking place more than six months before the diagnosis of SLE, (b) SLE cofounders more likely to cause the event, and (c) meeting the “Ainala Criteria” (headaches, mild depression, anxiety, minor cognitive complaints, and electromyography-negative polyneuropathy) [3].

This interesting case not only stresses the importance of being aware of various causes of altered mental status before diagnosing patients with strictly psychiatric disorders, but it also makes the case for the presence of more than one etiology coexisting and causing the patient’s symptoms. Among these, drug overdose, alcohol intoxication, metabolic disturbances, medications with psychosis as a side effect, and trauma should be considered. It is worth noting that some patients with SLE might not meet the criteria for SLE diagnosis when the diagnosis is suspected, hence it is crucial to obtain an appropriate history including past symptoms and physical examination.

Our patient had an elevated gamma gap. However, immunoglobulins comprise the majority of proteins in states of viral infection, plasma cell malignancies, or autoimmune disease, explaining the elevation in total protein independent of albumin [1]. This gamma gap led to further investigation with our patient.

The SLICC is an international group that is dedicated to SLE clinical research. They revised the SLE diagnostic criteria to include different issues that had come up since the development of the 1982 criteria [2]. The new criteria require one of two combinations: (a) fulfillment of at least four out of 17 criteria, with at least one clinical criterion and one immunological criterion, or (b) biopsy-confirmed lupus nephritis as the sole clinical criterion in the presence of elevated ANA or anti-dsDNA antibodies. SLICC also takes into account additional lupus manifestations, such as chronic cutaneous lupus, and includes the anti-Smith antibody as an individual criterion. In terms of neurologic aspects of lupus, it now includes more manifestations than the original American College of Rheumatology definition of seizures and psychosis. In addition, the SLICC criteria include mononeuritis multiplex, myelitis, peripheral or cranial neuropathy (in absence of other known causes), and acute confusional states (after ruling out other causes, including metabolic disturbances, uremia, and drugs). Of note, a specific selection on neuropsychiatric manifestations was not included because they were considered not specific to SLE.

In 1999, the American College of Rheumatology made recommendations regarding NPSLE based on 19 neuropsychiatric conditions, including 12 from central nervous system (CNS) and seven from the peripheral nervous system (PNS) [3]. The CNS conditions include, but are not limited to, headache, seizure disorder, cerebrovascular disease, cognitive dysfunction, mood disorder, anxiety disorder, psychosis, and acute confusional state. On the other hand, PNS conditions include mononeuropathy, Guillain-Barre syndrome, and autonomic disorders, among others. There are different pathways involved in the development of each of the CNS cases. For example, vasculopathy has been linked to the presence of antiphospholipid (aPL) antibodies and other autoantibodies through a compromised blood-brain barrier. Another example was described in a 2014 meta-analysis suggesting that anti-ribosomal P antibodies are specifically related to psychosis in NPSLE. In this article, Kivity, et al. suggest that anti-ribosomal P antibodies bind to the neuronal surface-P antigen, penetrate neuronal cells, and inhibit protein synthesis. It was recently identified that anti-ribosomal P antibodies may interact with neuronal surface-P antigen on the surface of hippocampal neurons leading to neuronal apoptosis [4]. These antibodies specifically target the hippocampus and amygdala, which can explain their association with depression and cognitive dysfunction. Anti-dsDNA antibodies, well known to be involved in the SLE disease inflammatory process, have been shown to recognize a specific sequence in the N-methyl-D-aspartate receptors NR2a and NR2b [3]. The anti-dsDNA and anti-NR2 antibodies are detected in serum and CSF in approximately 25-50% of SLE patients and there have been associations between their levels and NPSLE symptoms, specifically mood disorders, acute confusional state, and cognitive decline. Anti-NR2 antibodies have been found in very high levels in the CSF, as well as significant blood-brain barrier (BBB) damage in a patient with severe diffuse NPSLE (acute confusional state). BBB damage is highly related to proinflammatory cytokine production, especially interleukin-6 (IL-6) and IL-8, and interferon (IFN)-alpha and gamma, leading to inflammation, allowing for barrier breach. There is also growing evidence regarding complement C5a causing BBB dysfunction by inducing pro-inflammatory cytokines by way of reactive oxygen species and actin reorganization. CSF analysis with cell count between 100-300 cells per mm3, predominantly lymphocytes, can signal SLE [5].

Once other causes of AMS are excluded and the diagnosis of NPSLE has been established, it should be treated with immunosuppressive therapy. Steroids are first-line drugs (methylprednisolone 500 mg-1 g/day for 3-5 days, followed by prednisolone 1 mg/kg/day), and cyclophosphamide 1 g per month for six months. The six-month induction period is followed by quarterly maintenance doses for total of two years. Adjunctive treatment with antidepressants or antipsychotics may also be used [6,7]. Additionally, there have been studies supporting the use of rituximab and belimumab in severe NPSLE [4]. Rituximab, the monoclonal antibody against B-cell antigen CD-20, has been used specifically in refractory SLE because B-cells are key in the disease pathogenesis, as well as autoantibody, cytokine, and chemokine production. The dose typically used is 375 mg/m2 weekly (x2) or 1 g 2 weeks apart. For cases that do not respond to the above treatments, intravenous immunoglobulins from healthy donors may be an alternative option, however, this is only based on case reports, case series, and retrospective studies. More randomized controlled trials are needed to support its use [7].

We recommend SLE to be kept in the list of differential diagnoses when a patient presents with AMS without a clear etiology. Furthermore, we recommend that neuropsychiatric lupus be considered when a patient with prior diagnosis of lupus presents with a change in mental status. By starting the workup with simple tests, one should aim to rule out lupus by ordering serum ANA and measuring a random protein/creatinine ratio if renal compromise is suspected. In cases with indeterminate diagnosis by SLICC criteria and known nephritic proteinuria, which can change the course of possible treatment, it is important to obtain a renal biopsy. Lupus nephritis is known as one of the “stand-alone” diagnostic criteria for SLE. In summary, the SLICC criteria and a strong clinical acumen can help guide clinicians when the final diagnosis is unclear before other, more invasive tests are performed.

## Conclusions

The distinction between neuropsychiatric systemic lupus erythematosus, formerly known as lupus cerebritis, and functional psychological disorders remains a diagnostic dilemma. Also, the varied diagnostic criteria of these manifestations make the diagnosis even more complex. Hence, in a patient presenting with altered mental status of unexplained etiology, high clinical suspicion for Lupus along with detailed history, and workup led by the SLICC criteria can help clinicians solve this mystery.
